# Integrating Non-monotonic Logical Reasoning and Inductive Learning With Deep Learning for Explainable Visual Question Answering

**DOI:** 10.3389/frobt.2019.00125

**Published:** 2019-12-11

**Authors:** Heather Riley, Mohan Sridharan

**Affiliations:** ^1^Electrical and Computer Engineering, The University of Auckland, Auckland, New Zealand; ^2^Intelligent Robotics Lab, School of Computer Science, University of Birmingham, Birmingham, United Kingdom

**Keywords:** nonmonotonic logical reasoning, inductive learning, deep learning, visual question answering, commonsense reasoning, human-robot collaboration

## Abstract

State of the art algorithms for many pattern recognition problems rely on data-driven deep network models. Training these models requires a large labeled dataset and considerable computational resources. Also, it is difficult to understand the working of these learned models, limiting their use in some critical applications. Toward addressing these limitations, our architecture draws inspiration from research in cognitive systems, and integrates the principles of commonsense logical reasoning, inductive learning, and deep learning. As a motivating example of a task that requires explainable reasoning and learning, we consider Visual Question Answering in which, given an image of a scene, the objective is to answer explanatory questions about objects in the scene, their relationships, or the outcome of executing actions on these objects. In this context, our architecture uses deep networks for extracting features from images and for generating answers to queries. Between these deep networks, it embeds components for non-monotonic logical reasoning with incomplete commonsense domain knowledge, and for decision tree induction. It also incrementally learns and reasons with previously unknown constraints governing the domain's states. We evaluated the architecture in the context of datasets of simulated and real-world images, and a simulated robot computing, executing, and providing explanatory descriptions of plans and experiences during plan execution. Experimental results indicate that in comparison with an “end to end” architecture of deep networks, our architecture provides better accuracy on classification problems when the training dataset is small, comparable accuracy with larger datasets, and more accurate answers to explanatory questions. Furthermore, incremental acquisition of previously unknown constraints improves the ability to answer explanatory questions, and extending non-monotonic logical reasoning to support planning and diagnostics improves the reliability and efficiency of computing and executing plans on a simulated robot.

## 1. Introduction

Deep neural network architectures and the associated algorithms represent the state of the art for many perception and control problems in which their performance often rivals that of human experts. These architectures and algorithms are increasingly being used for a variety of tasks such as object recognition, gesture recognition, object manipulation, and obstacle avoidance, in domains such as healthcare, surveillance, and navigation. Common limitations of deep networks are that they are computationally expensive to train, and require a large number of labeled training samples to learn an accurate mapping between input(s) and output(s) in complex domains. It is not always possible to satisfy these requirements, especially in dynamic domains where previously unseen situations often change the mapping between inputs and outputs over time. Also, it is challenging to understand or provide an explanatory description of the observed behavior of a learned deep network model. Furthermore, it is difficult to use domain knowledge to improve the computational efficiency of learning these models or the reliability of the decisions made by these models. Consider a self-driving car on a busy road. Any error made by the car, e.g., in recognizing or responding to traffic signs, can result in serious accidents and make humans more reluctant to use such cars. In general, it is likely that humans interacting with a system designed for complex domains, with autonomy in some components, will want to know why and how the system arrived at particular conclusions; this “explainability” will help designers improve the underlying algorithms and their performance. Understanding the operation of these systems will also help human users build trust in the decisions made by these systems. Despite considerable research in recent years, providing explanatory descriptions of decision making and learning continues to be an open problem in AI.

We consider Visual Question Answering (VQA) as a motivating example of a complex task that inherently requires explanatory descriptions of reasoning and learning. Given a scene and a natural language question about an image of the scene, the objective of VQA is to provide an accurate answer to the question. These questions can be about the presence or absence of particular objects in the image, the relationships between these objects, or the potential outcome of executing particular actions on objects in the scene. For instance, a system recognizing and responding to traffic signs on a self-driving car may be posed questions such as “what is the traffic sign in the image?,” or “what is the meaning of this traffic sign?,” and a system controlling a robot arm constructing stable arrangements of objects on a tabletop may be asked “why is this structure unstable?” or “what would make the structure stable?” We assume that any such questions are provided as (or transcribed into) text, and that answers to questions are also generated as text (that may be converted to speech) using existing software. Deep networks represent the state of the art for VQA, but are characterized by the known limitations described above. We seek to address these limitations by drawing inspiration from research in cognitive systems, which indicates that reliable, efficient, and explainable reasoning and learning can be achieved in complex problems by jointly reasoning with commonsense domain knowledge and learning from experience. Specifically, the architecture described in this paper tightly couples knowledge representation, reasoning, and learning, and exploits the complementary strengths of deep learning, inductive learning, and non-monotonic logical reasoning with incomplete commonsense domain knowledge. We describe the following characteristics of the architecture:

- For any input image of a scene of interest, Convolutional Neural Networks (CNNs) extract concise visual features characterizing the image.
- Non-monotonic logical reasoning with the extracted features and incomplete commonsense domain knowledge is used to classify the input image, and to provide answers to explanatory questions about the classification and the scene.
- Feature vectors that the non-monotonic logical reasoning is unable to classify are used to train a decision tree classifier that is also used to answer questions about the classification during testing.
- Feature vectors not classified by non-monotonic logical reasoning, along with the output of the decision tree classifier, train a Recurrent Neural Network (RNN) that is used to answer explanatory questions about the scene during testing.
- Feature vectors not classified by non-monotonic logical reasoning are also used to inductively learn, and subsequently reason with, constraints governing domain states; and
- Reasoning with commonsense knowledge is expanded (when needed) to support planning, diagnostics, and the ability to answer related explanatory questions.

This architecture builds on our prior work on combining commonsense inference with deep learning (Riley and Sridharan, 2018a; Mota and Sridharan, 2019) by introducing the ability to learn and reason with constraints governing domain states, and extending explainable inference with commonsense knowledge to also support planning and diagnostics to achieve any given goal.

Although we use VQA as a motivating example, it is not the main focus of our work. State of the art algorithms for VQA focus on generalizing to images from different domains, and are evaluated on benchmark datasets of several thousand images drawn from different domains (Shrestha et al., 2019). Our focus, on the other hand, is on transparent reasoning and learning in any given domain in which a large, labeled dataset is not readily available. Toward this objective, our approach explores the interplay between non-monotonic logical reasoning, incremental inductive learning, and deep learning. We thus neither compare our architecture and algorithms with state of the art algorithms for VQA, nor use large benchmark VQA datasets for evaluation. Instead, we evaluate our architecture's capabilities in the context of: (i) estimating the stability of configurations of simulated blocks on a tabletop; (ii) recognizing different traffic signs in a benchmark dataset of images; and (iii) a simulated robot delivering messages to the intended recipients at different locations. The characteristics of these tasks and domains match our objective. In both domains, we focus on answering explanatory questions about images of scenes and the underlying classification problems (e.g., recognizing traffic signs). In addition, we demonstrate how our architecture can be adapted to enable a robot assisting humans to compute and execute plans, and to answer questions about these plans. Experimental results show that in comparison with an architecture based only on deep networks, our architecture provides: (i) better accuracy on classification problems when the training dataset is small, and comparable accuracy on larger datasets; and (ii) significantly more accurate answers to explanatory questions about the scene. We also show that the incremental acquisition of state constraints improves the ability to answer explanatory questions, and to compute minimal and correct plans.

We begin with a discussion of related work in section 2. The architecture and its components are described in section 3, with the experimental results discussed in section 4. Section 5 then describes the conclusions and directions for further research.

## 2. Related Work

State of the art approaches for VQA are based on deep learning algorithms (Jiang et al., 2015; Masuda et al., 2016; Malinowski et al., 2017; Pandhre and Sodhani, 2017; Zhang et al., 2017; Shrestha et al., 2019). These algorithms use labeled data to train neural network architectures with different arrangements of layers and connections between them, capturing the mapping between the inputs (e.g., images, text descriptions) and the desired outputs (e.g., class labels, text descriptions). Although deep networks have demonstrated the ability to model complex non-linear mappings between inputs and outputs for different pattern recognition tasks, they are computationally expensive and require large, labeled training datasets. They also make it difficult to understand and explain the internal representations, identify changes that will improve performance, or to transfer knowledge acquired in one domain to other related domains. In addition, it is challenging to accurately measure performance or identify dataset bias, e.g., deep networks can answer questions about images using question-answer training samples without even reasoning about the images (Jabri et al., 2016; Teney and van den Hengel, 2016; Zhang et al., 2017). There is on-going research on each of these issues, e.g., to explain the operation of deep networks, reduce training data requirements and bias, reason with domain knowledge, and incrementally learn the domain knowledge. We review some of these approaches below, primarily in the context of VQA.

Researchers have developed methods to understand the internal reasoning of deep networks and other machine learning algorithms. Selvaraju et al. (2017) use the gradient in the last convolutional layer of a CNN to compute the relative contribution (importance weight) of each neuron to the classification decision made. However, the weights of neurons do not provide an intuitive explanation of the CNN's operation or its internal representation. Researchers have also developed general approaches for understanding the predictions of any given machine learning algorithm. For instance, Koh and Liang (2017) use second-order approximations of influence diagrams to trace any model's prediction through a learning algorithm back to the training data in order to identify training samples most responsible for any given prediction. Ribeiro et al. (2016) developed a framework that analyzes any learned classifier model by constructing a interpretable simpler model that captures the essence of the learned model. This framework formulates the task of explaining the learned model, based on representative instances and explanations, as a submodular optimization problem. In the context of VQA, Norcliffe-Brown et al. (2018) provide interpretability by introducing prior knowledge of scene structure as a graph that is learned from observations based on the question under consideration. Object bounding boxes are graph nodes while edges are learned using an attention model conditioned on the question. Mascharka et al. (2018) augment a deep network architecture with an image-space attention mechanism based on a set of composable visual reasoning primitives that help examine the intermediate outputs of each module. Li et al. (2018) introduce a captioning model to generate an image's description, reason with the caption and the question to construct an answer, and use the caption to explain the answer. However, these algorithms do not support the use of commonsense reasoning to (i) provide meaningful explanatory descriptions of learning and reasoning; (ii) guide learning to be more efficient; or (iii) provide reliable decisions when large training datasets are not available.

The training data requirements of a deep network can be reduced by directing attention to data relevant to the tasks at hand. In the context of VQA, Yang et al. (2016) use a Long Short-Term Memory (LSTM) network to map the question to an encoded vector, extract a feature map from the input image using a CNN, and use a neural network to compute weights for feature vectors based on their relevance to the question. A stacked attention network is trained to map the weighted feature vectors and question vector to the answer, prioritizing feature vectors with greater weights. Schwartz et al. (2017) use learned higher-order correlations between various data modalities to direct attention to elements in the data modalities that are relevant to the task at hand. Lu et al. (2016) use information from the question to identify relevant image regions and uses information from the image to identify relevant words in the question. A co-attentional model jointly and hierarchically reasons about the image and the question at three levels, embedding words in a vector space, using one-dimensional CNNs to model information at the phrase level, and using RNNs to encode the entire question. A generalization of this work, a Bilinear Attention Network, considers interactions between all region proposals in the image with all words in the (textual) question (Kim et al., 2018). A Deep Attention Neural Tensor Network for VQA, on the other hand, uses tensor-based representations to discover joint correlations between images, questions, and answers (Bai et al., 2018). The attention module is based on a discriminative reasoning process, and regression with KL-divergence losses improves scalability of training and convergence. Recent work by Anderson et al. (2018) combines top-down and bottom-up attention mechanisms, with the top-down mechanism providing an attention distribution over object proposals provided by the bottom-up mechanism.

In addition to reducing the training data requirements, researchers have focused on reducing the number of annotated samples needed for training, and on minimizing the bias in deep network models. In the context of VQA, Lin et al. (2014) iteratively revise a model trained on an initial training set by expanding the training set with image-question pairs involving concepts it is uncertain about, with an “oracle” (human annotater) providing the answers. This approach reduces annotation time, but the database includes just as many images and questions as before. Goyal et al. (2017) provide a balanced dataset with each question associated with a pair of images that require different answers, and provide a counterexample based explanation for each image-question pair. Agrawal et al. (2018), on the other hand, separate the recognition of visual concepts in an image from the identification of an answer to any given question, and include inductive biases to prevent the learned model from relying predominantly on priors in the training data.

In computer vision, robotics and other applications, learning from data can often be made more efficient by reasoning with prior knowledge about the domain. In the context of VQA, Wang et al. (2017) reason with knowledge about scene objects to answer common questions about these objects, significantly expanding the range of natural language questions that can be answered without making the training data requirements impractical. However, this approach does not reduce the amount of data required to train the deep network. Furbach et al. (2010) directly use a knowledge base to answer questions and do not consider the corresponding images as inputs. Wagner et al. (2018), on the other hand, use physics engines and prior knowledge of domain objects to realistically simulate and explore different situations. These simulations guide the training of deep network models that anticipate action outcomes and answer questions about all situations. Based on the observation that VQA often requires reasoning over multiple steps, Wu et al. (2018) construct a chain of reasoning for multi-step and dynamic reasoning with relations and objects. This approach iteratively forms new relations between objects using relational reasoning operations, and forms new compound objects using object refining operations, to improve VQA performance. Given the different components of a VQA system, Teney and van den Hengel (2018) present a meta learning approach to separate question answering from the information required for the task, reasoning at test time over example questions and answers to answer any given question. Two meta learning methods adapt a VQA model without the need for retraining, and demonstrate the ability to provide novel answers and support vision and language learning. Rajani and Mooney (2018) developed an ensemble learning approach, Stacking With Auxiliary Features, which combines the results of multiple models using features of the problem as context. The approach considers four categories of auxiliary features, three of which are inferred from image-question pairs while the fourth uses model-specific explanations.

Research in cognitive systems indicates that reliable, efficient, and explainable reasoning and learning can be achieved by reasoning with domain knowledge and learning from experience. Early work by Gil (1994) enabled an agent to reason with first-order logic representations and incrementally refined action operators. In such methods, it is difficult to perform non-monotonic reasoning, or to merge new, unreliable information with existing beliefs. Non-monotonic logic formalisms have been developed to address these limitations, e.g., Answer Set Prolog (ASP) has been used in cognitive robotics (Erdem and Patoglu, 2012) and other applications (Erdem et al., 2016). ASP has been combined with inductive learning to monotonically learn causal laws (Otero, 2003), and methods have been developed to learn and revise domain knowledge represented as ASP programs (Balduccini, 2007; Law et al., 2018). Cognitive architectures have also been developed to extract information from perceptual inputs to revise domain knowledge represented in first-order logic (Laird, 2012), and to combine logic and probabilistic representations to support reasoning and learning in robotics (Zhang et al., 2015; Sarathy and Scheutz, 2018). However, approaches based on classical first-order logic are not expressive enough, e.g., modeling uncertainty by attaching probabilities to logic statements is not always meaningful. Logic programming methods, on the other hand, do not support one or more of the desired capabilities such as efficient and incremental learning of knowledge, reasoning efficiently with probabilistic components, or generalization as described in this paper. These challenges can be addressed using interactive task learning, a general knowledge acquisition framework that uses labeled examples or reinforcement signals obtained from observations, demonstrations, or human instructions (Laird et al., 2017; Chai et al., 2018). Sridharan and Meadows (2018) developed such a framework to combine non-monotonic logical reasoning with relational reinforcement learning and inductive learning to learn action models to be used for reasoning or learning in dynamic domains. In the context of VQA, there has been interesting work on reasoning with learned symbolic structure. For instance, Yi et al. (2018) present a neural-symbolic VQA system that uses deep networks to infer structural object-based scene representation from images, and to generate a hierarchical (symbolic) program of functional modules from the question. An executor then runs the program on the representation to answer the question. Such approaches still do not (i) integrate reasoning and learning such that they inform and guide each other; or (ii) use the rich domain-specific commonsense knowledge that is available in any application domain.

In summary, deep networks represent the state of the art for VQA and many other pattern recognition tasks. Recent surveys on VQA methods indicate that despite considerable research, it is still difficult to use these networks to support efficient learning, intuitive explanations, or generalization to simulated and real-world images (Pandhre and Sodhani, 2017; Shrestha et al., 2019). Our architecture draws on principles of cognitive systems to address these limitations. It tightly couples deep networks with components for non-monotonic logical reasoning with commonsense domain knowledge, and for learning incrementally from samples over which the learned model makes errors. This work builds on our proof of concept architecture that integrated deep learning with commonsense inference for VQA (Riley and Sridharan, 2018a). It also builds on work in our research group on using commonsense inference and learned state constraints to guide deep networks that estimate object stability and occlusion in images (Mota and Sridharan, 2019). In comparison with our prior work, we introduce a new component for incrementally learning constraints governing domain states, expand reasoning with commonsense knowledge to support planning and diagnostics, explore the interplay between the architecture's components, and discuss detailed experimental results.

## 3. Architecture

**Figure 1:**
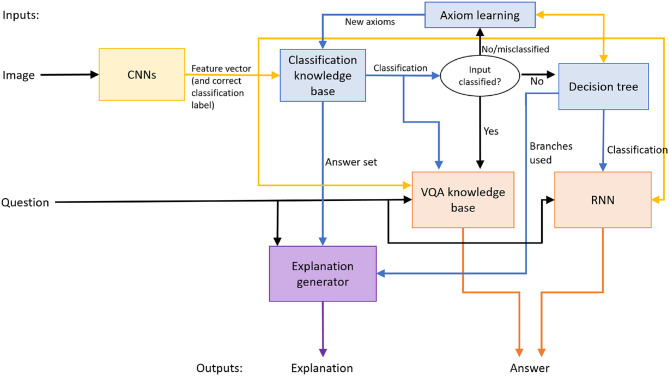
Architecture combines the complementary strengths of deep learning, non-monotonic logical reasoning with commonsense domain knowledge, and decision-tree induction.

Figure 1 is an overview of our architecture that provides answers to explanatory questions about images of scenes and an underlying classification problem. The architecture seeks to improve accuracy and reduce training effort, i.e., reduce training time and the number of training samples, by embedding non-monotonic logical reasoning and inductive learning in a deep network architecture. We will later demonstrate how the architecture can be adapted to address planning problems on a simulated robot—see section 3.5. The architecture may be viewed as having four key components that are tightly coupled with each other.

1. A component comprising CNN-based feature extractors, which are trained and used to map any given image of a scene under consideration to a vector of image features.
2. A component that uses one of two methods to classify the feature vector. The first method uses non-monotonic reasoning with incomplete domain knowledge and the features to assign a class label and explain this decision. If the first method cannot classify the image, the second method trains and uses a decision tree to map the feature vector to a class label and explain the classification.
3. A component that answers explanatory questions. If non-monotonic logical reasoning is used for classification, it is also used to provide answers to these questions. If a decision tree is instead used for classification, an RNN is trained to map the decision tree's output, the image features, and the question, to the corresponding answer.
4. A component that uses the learned decision tree and the existing knowledge base to incrementally construct and validate constraints on the state of the domain. These constraints revise the existing knowledge that is used for subsequent reasoning.

This architecture exploits the complementary strengths of deep learning, non-monotonic logical reasoning, and incremental inductive learning with decision trees. Reasoning with commonsense knowledge guides learning, e.g., the RNN is trained on (and processes) input data that cannot be processed using existing knowledge. The CNNs and RNN can be replaced by other methods for extracting image features and answering explanatory questions (respectively). Also, although the CNNs and RNN are trained in an initial phase in this paper, these models can be revised over time if needed. We hypothesize that embedding non-monotonic logical reasoning with commonsense knowledge and the incremental updates of the decision tree, between the CNNs and the RNN, makes the decisions more transparent, and makes learning more time and sample efficient. Furthermore, the overall architecture and methodology can be adapted to different domains. In this paper, we will use the following two domains to illustrate and evaluate the architecture's components and the methodology.

**Figure 2:**
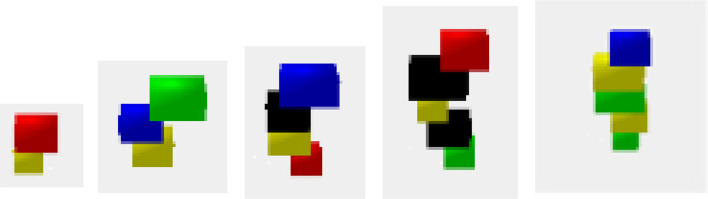
Illustrative images of structures of blocks of different colors and sizes; these images were obtained from a physics-based simulator for the SS domain.

**Figure 3:**
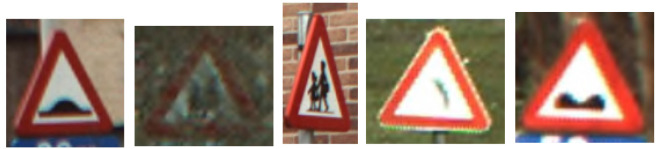
Illustrative images of traffic signs from the BelgiumTS dataset (Timofte et al., 2013).

1. **Structure Stability (SS):** this domain has different structures, i.e., different arrangements of simulated blocks of different colors and sizes, on a tabletop—see Figure 2 for some examples. We generated 2,500 such images using a physics-based simulator. The relevant features of the domain include the number of blocks, whether the structure is on a lean, whether the structure has a narrow base, and whether any block is placed such that it is not well balanced on top of the block below. The objective in this domain is to classify structures as being stable or unstable, and to answer explanatory questions such as “why is this structure unstable?” and “what should be done to make this structure stable?”
2. **Traffic Sign (TS):** this domain focuses on recognizing traffic signs from images—see Figure 3 for some examples. We used the BelgiumTS benchmark dataset (Timofte et al., 2013) with ≈ 7000 real-world images (total) of 62 different traffic signs. This domain's features include the primary symbol of the traffic sign, the secondary symbol, the shape of the sign, the main color in the middle, the border color, the sign's background image, and the presence or absence of a cross (e.g., some signs have a red or black cross across them to indicate the end of a zone, with the absence of the cross indicating the zone's beginning). The objective is to classify the traffic signs and answer explanatory questions such as “what is the sign's message?” and “how should the driver respond to this sign?”

In addition to these two domains, section 3.5 will introduce the **Robot Assistant (RA)** domain, a simulated domain to demonstrate the use of our architecture for computing and executing plans to achieve assigned goals. In the *RA* domain, a simulated robot reasons with existing knowledge to deliver messages to target people in target locations, and to answer explanatory questions about the plans and observed scenes.

The focus of our work is on understanding and using the interplay between deep learning, commonsense reasoning, and incremental learning, in the context of *reliable and efficient scene understanding in any given dynamic domain*. The benchmark VQA datasets and the algorithms, on the other hand, focus on generalizing across images from different scenarios in different domains, making it difficult to support the reasoning and learning capabilities of our architecture. We thus do not use these datasets or algorithms in our evaluation.

### 3.1. Feature Extraction Using CNNs

The first component of the architecture trains CNNs to map input images to concise features representing the objects of interest in the images. For the SS domain and TS domain, semi-automated annotation was used to label the relevant features in images for training and testing. The selection of these features for each domain was based on domain expertise. In the SS domain, the features of interest are:

- Number of blocks in structure (number ∈ [1, 5]);
- Whether the structure is on a lean (true, false);
- Width of the base block (wide, narrow); and
- Whether any block is displaced, i.e., not well balanced on top of the block below (true, false).

In the TS domain, the features of interest are:

- Primary symbol in the middle of the traffic sign; 39 primary symbols such as *bumpy*_*road*, *slippery*_*road*, *stop*, *left*_*turn*, and *speed*_*limit*;
- Secondary symbol in the traffic sign; 10 secondary symbols such as *disabled*, *car* and *fence*;
- Shape of the sign; *circle*, *triangle*, *square*, *hexagon*, *rectangle*, *widerectangle*, *diamond*, or *inverted* *triangle*;
- Main color in the middle of the sign; *red*, *white*, or *blue*;
- Border color at the edge of the sign; *red*, *white*, or *blue*;
- Background image, e.g., some symbols are placed over a square or a triangle; and
- Presence of a red or black cross across a sign to indicate a zone's end or invalidity; the sign without the cross indicates the zone's beginning or validity, e.g., a parking sign with a cross implies no parking.

**Figure 4:**
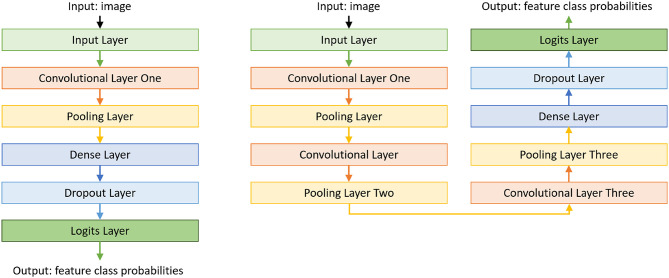
Basic CNN model used for extracting each feature in our architecture. CNNs for individual features may end up with a different number of convolutional layers and pooling layers.

To reduce the training data requirements and simplify the training of CNNs, we (i) train a separate CNN for each feature to be extracted from an image; and (ii) start with a basic model for each CNN and incrementally make it more complex as needed. The number of CNNs is thus equal to the number of features to be extracted from each image for any given domain, and the CNN trained for each feature may be different even within a particular domain. The basic CNN model we begin with has an input layer, a convolutional layer, a pooling layer, a dense layer, a dropout layer, and a logit layer, as seen on the left of Figure 4. Additional convolutional and pooling layers are added until the feature extraction accuracy converges or exceeds a threshold (e.g., ≥ 90%). Our architecture also includes the option of fine-tuning previously trained CNN models instead of starting from scratch. The right side of Figure 4 shows a CNN model learned in our example domains, which has three convolutional layers and pooling layers. We trained and validated these CNNs in an initial phase, and used them for evaluation. Our code for constructing these CNNs for features (in our example domains) is in our repository (Riley and Sridharan, 2018b).

### 3.2. Classification Using Non-monotonic Logical Reasoning or Decision Trees

The feature vector extracted from an image is used for decision making. In the SS domain and TS domain, decisions take the form of assigning a class label to each feature vector^1^. The second component of our architecture performs this task using one of two methods: (i) non-monotonic logical inference using ASP; or (ii) a classifier based on a learned decision tree. We describe these two methods below.

#### 3.2.1. ASP-Based Inference With Commonsense Knowledge

The first step in reasoning with incomplete commonsense domain knowledge is the representation of this knowledge. In our architecture, an *action language* is used to describe the dynamics of any domain under consideration. Action languages are formal models of parts of natural language used for describing transition diagrams of dynamic systems. Our architecture uses action language ${\mathrm{AL}}_{d}$ (Gelfond and Inclezan, 2013), with a *sorted signature* Σ that can be viewed as the *vocabulary* used to describe the domain's transition diagram. The signature Σ comprises *basic sorts*, which are similar to *types* in a programming language, *statics*, i.e., domain attributes whose values do not change over time, *fluents*, i.e., domain attributes whose values can change over time, and *actions*. The domain's fluents can be *basic*, i.e., those that obey the laws of inertia and are changed directly by actions, or *defined*, i.e., those that do not obey the laws of inertia and are defined by other attributes. A domain attribute or its negation is a *literal*; of all its variables are ground, it is a *ground literal*. ${\mathrm{AL}}_{d}$ allows three types of statements: *causal law, state constraint* and *executability condition*.

$$\begin{array}{llll} \;a\;causes\;l_{b} & if & p_{0},\ldots ,p_{m} & \left(\text{Causal law}\right)\\ \;l & if & p_{0},\ldots ,p_{m} & \left(\text{State constraint}\right)\\ impossible\;a_{0},\ldots ,a_{k} & if & p_{0},\ldots ,p_{m} & \left(\text{Executability condition}\right) \end{array}$$

where *a* is an action, *l* is a literal, *l*_*b*_ is a basic literal, and *p*_0_, …, *p*_*m*_ are domain literals.

The domain representation (i.e., the knowledge base) comprises a *system description* D, which is a set of statements of ${\mathrm{AL}}_{d}$, and a *history* H. D comprises a sorted signature Σ and axioms describing the domain dynamics. For instance, in the SS domain, Σ includes basic sorts such as *structure*, *color*, *size*, and *attribute*; the basic sorts of the TS domain include *main*_*color*, *other*_*color*, *main*_*symbol*, *other*_*symbol*, *shape*, *cross* etc. The sort *step* is also in Σ to support temporal reasoning over time steps. The statics and fluents in the SS domain include:

(1) $$\begin{array}{l} num\text{\_}blocks\left(structure,num\right),block\text{\_}color\left(block,color\right),\\ block\text{\_}size\left(block,size\right)block\text{\_}displaced\left(structure\right),stable\left(structure\right) \end{array}$$

which correspond to the image features extracted in the domain, and are described in terms of their arguments' sorts. In a similar manner, statics and fluents of the TS domain include:

(2) $$\begin{array}{l} primary\text{\_}symbol\left(sign,main\text{\_}symbol\right),\\ primary\text{\_}color\left(sign,main\text{\_}color\right)\\ secondary\text{\_}symbol\left(sign,other\text{\_}symbol\right),\\ secondary\text{\_}color\left(sign,other\text{\_}color\right)\\ sign\text{\_}shape\left(shape\right),background\text{\_}image\left(image\right) \end{array}$$

In both domains, signature Σ includes a predicate *holds*(*fluent, step*), which implies that a particular fluent holds true at a particular time step. As stated above, Σ for a dynamic domain typically includes actions that cause state transitions, but this capability is not needed to answer explanatory questions about specific scenes and the underlying classification problem in our (SS, TS) domains. For ease of explanation, we thus temporarily disregard the modeling of actions, and their preconditions and effects. We will revisit actions in section 3.5 when we consider planning tasks in the RA domain.

Given a signature Σ for a domain, a *state* of the domain is a collection of ground literals, i.e., statics, fluents, actions and relations with values assigned to their arguments—for more details, please see Gelfond and Kahl (2014) and Sridharan et al. (2019). The axioms of D are defined in terms of the signature and govern domain dynamics; this typically includes a distributed representation of the constraints related to domain actions, i.e., causal laws and executability conditions that define the preconditions and effects of actions, and constraints related to states, i.e., state constraints. In the SS domain and TS domain, axioms govern the belief about domain states; we will discuss axioms related to actions in section 3.5 when we discuss the RA domain. Specifically, the axioms of the SS domain include state constraints such as:

(3a) $$\begin{array}{l} \neg stable\left(S\right)\;\text{i}\text{f}\;block\text{\_}displaced\left(S\right) \end{array}$$

(3b) $$\begin{array}{l} \;stable\left(S\right)\;\text{i}\text{f}\;num\text{\_}blocks\left(S,2\right),\;\neg structure\text{\_}type\left(S,lean\right) \end{array}$$

where Statement 3(a) says that any structure with a block that is displaced significantly is unstable, and Statement 3(b) says that any pair of blocks without a significant lean is stable.

Axioms of the TS domain include statements such as:

(4a) $$\begin{array}{l} sign\text{\_}type\left(TS,no\text{\_}parking\right)\;\text{i}\text{f}\;primary\text{\_}color\left(TS,blue\right),\\ \;primary\text{\_}symbol\left(TS,blank\right),\\ \;cross\left(TS\right),shape\left(TS,circle\right) \end{array}$$

(4b) $$\begin{array}{l} \;sign\text{\_}type\left(TS,stop\right)\;\text{i}\text{f}\;primary\text{\_}color\left(TS,red\right),\\ \;primary\text{\_}symbol\left(TS,stoptext\right),\\ \;shape\left(TS,octagon\right) \end{array}$$

where Statement 4(a) implies that a blue, blank, circular traffic sign with a cross across it is a no parking sign. Statement 4(b) implies that a red, octagon-shaped traffic sign with the text “stop” is a stop sign.

The history H of a dynamic domain is usually a record of fluents observed to be true or false at a particular time step, i.e., *obs*(*fluent, boolean, step*), and the successful execution of an action at a particular time step, i.e., *hpd*(*action, step*); for more details, see Gelfond and Kahl (2014). The domain knowledge in many domains often includes default statements that are true in all but a few exceptional circumstances. For example, we may know in the SS domain that “structures with two blocks of the same size are usually stable.” To encode such knowledge, we use our recent work that expanded the notion of history to represent and reason with defaults describing the values of fluents in the initial state (Sridharan et al., 2019).

Key tasks of an agent equipped with a system description D and history H include reasoning with this knowledge for inference, planning and diagnostics. In our architecture, these tasks are accomplished by translating the domain representation to a program Π(D,H) in CR-Prolog, a variant of ASP that incorporates consistency restoring (CR) rules (Balduccini and Gelfond, 2003). In this paper, we use the terms “ASP” and “CR-Prolog” interchangeably. ASP is a declarative programming paradigm designed to represent and reason with incomplete commonsense domain knowledge. It is based on stable model semantics, and supports *default negation* and *epistemic disjunction*. For instance, unlike “¬*a*”, which implies that *a is believed to be false*, “*nota*” only implies *a is not believed to be true*. Also, unlike “*p* ∨ ¬*p*” in propositional logic, “*p or* ¬*p*” is not tautological. Each literal can thus be true, false or unknown, and the agent reasoning with domain knowledge does not believe anything that it is not forced to believe. ASP can represent recursive definitions, defaults, causal relations, special forms of self-reference, and language constructs that occur frequently in non-mathematical domains, and are difficult to express in classical logic formalisms (Baral, 2003; Gelfond and Kahl, 2014). Unlike classical first-order logic, ASP supports non-monotonic logical reasoning, i.e., it can revise previously held conclusions or equivalently reduce the set of inferred consequences, based on new evidence—this ability helps the agent recover from any errors made by reasoning with incomplete knowledge. ASP and other paradigms that reason with domain knowledge are often criticized for requiring considerable (if not complete) prior knowledge and manual supervision, and for being unwieldy in large, complex domains. However, modern ASP solvers support efficient reasoning in large knowledge bases with incomplete knowledge, and are used by an international research community for cognitive robotics (Erdem and Patoglu, 2012; Zhang et al., 2015) and other applications (Erdem et al., 2016). For instance, recent work has demonstrated that ASP-based non-monotonic logical reasoning can be combined with: (i) probabilistic reasoning for reliable and efficient planning and diagnostics (Sridharan et al., 2019); and (ii) relational reinforcement learning and active learning methods for interactively learning or revising commonsense domain knowledge based on input from sensors and humans (Sridharan and Meadows, 2018).

In our architecture, the automatic translation from statements in ${\mathrm{AL}}_{d}$ to the program Π is based on a custom-designed script^2^. The resultant program Π includes the signature and axioms of D, inertia axioms, reality checks, closed world assumptions for defined fluents and actions, and observations, actions, and defaults from H. For instance, Statements 3(a-b) are translated to:

(5a) $$\begin{array}{l} \neg stable\left(S\right)\;\leftarrow \;block\text{\_}displaced\left(S\right) \end{array}$$

(5b) $$\begin{array}{l} stable\left(S\right)\;\leftarrow \;num\text{\_}blocks\left(S,2\right),\;\neg structure\text{\_}type\left(S,lean\right) \end{array}$$

In addition, features extracted from an input image (to be processed) are encoded as the initial state of the domain in Π. Each *answer set* of Π(D,H) then represents the set of beliefs of an agent associated with this program. Algorithms for computing entailment, and for planning and diagnostics, reduce these tasks to computing answer sets of CR-Prolog programs. We compute answer sets of CR-Prolog programs using the SPARC system (Balai et al., 2013). The CR-Prolog programs for our example domains are in our open-source software repository (Riley and Sridharan, 2018b). For the classification task in our example domains, the relevant literals in the answer set provide the class label and an explanatory description of the assigned label (see section 3.3); we will consider the planning task in section 3.5. The accuracy of the inferences drawn from the encoded knowledge depends on the accuracy and extent of the knowledge encoded, but encoding comprehensive domain knowledge is difficult. The decision of what and how much knowledge to encode is made by the designer.

#### 3.2.2. Decision Tree Classifier

If ASP-based inference cannot classify the feature vector extracted from an image, the feature vector is mapped to a class label using a decision tree classifier learned from labeled training examples. In a decision tree classifier, each node is associated with a question about the value of a particular feature, with the child nodes representing the different answers to the question, i.e., the possible values of the feature. Each node is also associated with samples that satisfy the corresponding values of the features along the path from the root node to this node. We use a standard implementation of a decision tree classifier (Duda et al., 2000). This implementation uses the Gini measure to compute information gain (equivalently, the reduction in entropy) that would be achieved by splitting an existing node based on each feature that has not already been used to create a split in the tree. Among the features that provide a significant information gain, the feature that provides the maximum information gain is selected to split the node. If none of the features would result in any significant information gain, this node becomes a leaf node with a class label that matches a majority of the samples at the node.

**Figure 5:**
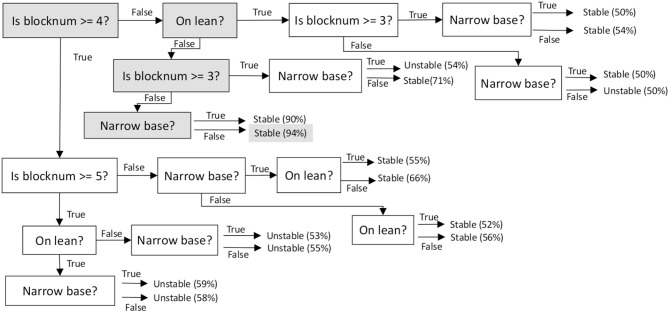
Example of part of a decision tree constructed from labeled samples and used for classification in the SS domain. The nodes used to classify a particular example are highlighted. Each leaf shows a class label and indicates the proportion of the labeled examples (at the leaf) that correspond to this label.

The decision tree's search space is quite specific since it only considers samples that could not be classified by ASP-based reasoning. The decision tree does not need to generalize as much as it would have to if it had to process every training (or test) sample in the dataset. Also, although overfitting is much less likely, we still use pruning to minimize the effects of overfitting. Figure 5 shows part of a learned decision tree classifier; specific nodes used to classify a particular example are highlighted to indicate that 94% of the observed examples of structures that have fewer than three blocks, do not have a significant lean, and do not have a narrow base, correspond to stable structures. These “active” nodes along any path in the decision tree that is used to classify an example can be used to explain the classification outcome in terms of the values of particular features that were used to arrive at the class label assigned to a specific image under consideration.

### 3.3. Answering Explanatory Questions

The third component of the architecture provides two methods for answering explanatory questions. The available inputs are the (i) question; (ii) vector of features extracted from the image under consideration; and (iii) classification output. The human designer also provides pre-determined templates for questions and their answers. In our case, we use a controlled vocabulary, templates based on language models and parts of speech for sentences, and existing software for natural language processing. Any given question is transcribed using the controlled vocabulary, parsed (e.g., to obtain parts of speech), and matched with the templates to obtain a relational representation. Recall that questions in the SS domain are of the form: “is this structure stable/unstable?” and “what is making this structure stable/unstable?” These questions can be translated into relational statements such as *stable*(*S*) or ¬*stable*(*S*) and used as a question, or as the desired consequence, during inference or in a search process. In a similar manner, questions in the TS domain such as: “what sign is this?” and “what is the sign's message?” can be translated into *sign*_*type*(*S, sign*) and used for subsequent processing.

The first method for answering explanatory questions is based on the understanding that if the feature vector extracted from the image is processed successfully using ASP-based reasoning, it is also possible to reason with the existing knowledge to answer explanatory questions about the scene. To support such question answering, we need to revise the signature Σ in the system description D of the domain. For instance, we add sorts such as *query*_*type*, *answer*_*type*, and *query* to encode different types of queries and answers. We also introduce suitable relations to represent questions, answers to these questions, and more abstract attributes, e.g., of structures of blocks, traffic signs etc.

In addition to the signature, we also augment the axioms in D to support reasoning with more abstract attributes, and to help construct answers to questions. For instance, we can include an axiom such as:

(6) $$\begin{array}{l} many\text{\_}blocks\left(S\right)\;\leftarrow \;unstable\left(S\right),\;\neg base\left(S,narrow\right),\\ \;\neg struc\text{\_}type\left(S,lean\right),\;\neg block\text{\_}displaced\left(S\right) \end{array}$$

which implies that if a structure (of blocks) is not on a narrow base, does not have a significant lean, and does not have blocks significantly displaced, any instability in the structure implies (and is potentially because) there are too many blocks in the structure. Once the ASP program Π(D,H) has been revised as described above, we can compute answer set(s) of this program to obtain the beliefs of the agent associated with this program. For any given question, the answer set(s) are parsed based on the known controlled vocabulary and templates (for questions and answers) to extract relevant literals—these literals are included in the corresponding templates to construct answers to explanatory questions. These answers can also be converted to speech using existing software.

**Figure 6:**
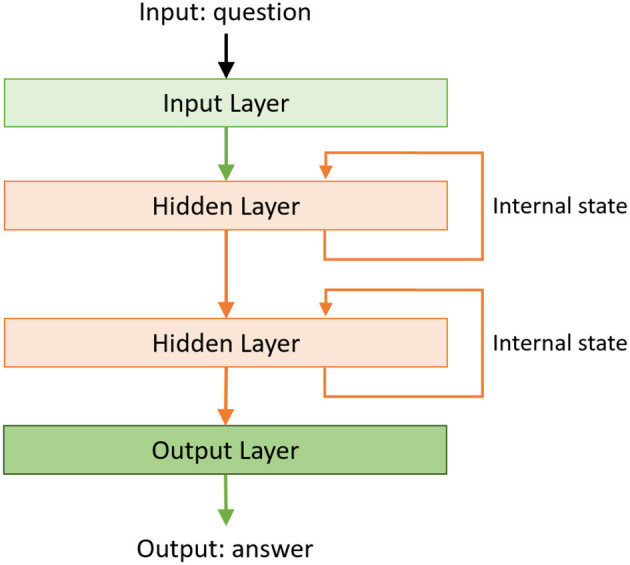
Example of the basic RNN used to construct explanations. The RNNs learned for the example domains considered in this paper have 26–30 hidden layers.

The second method for answering explanatory questions is invoked if the decision tree is used to process (i.e., classify in the context of the SS domain and TS domain) the vector of image features. The inability to classify the feature vector through ASP-based reasoning is taken to imply that the encoded domain knowledge is insufficient to answer explanatory questions about the scene. In this case, an LSTM network-based RNN is trained and used to answer the explanatory questions. The inputs are the feature vector, classification output, and a vector representing the transcribed and parsed query. The output (provided during training) is in the form of answers in the predetermined templates. Similar to the approach used in section 3.2, the RNN is built incrementally during training. We begin with one or two hidden layer(s), as shown in Figure 6, and add layers as long as it results in a significant increase in the accuracy. We also include the option of adding a stack of LSTMs if adding individual layers does not improve accuracy significantly. In our example domains, the RNN constructed to answer explanatory questions had as many as 26–30 hidden layers and used a softmax function to provide one of about 50 potential answer types. An example of the code used to train the RNN is available in our repository (Riley and Sridharan, 2018b).

### 3.4. Learning State Constraints

The components of the architecture described so far support reasoning with commonsense knowledge, learned decision trees, and deep networks, to answer explanatory questions about the scene and an underlying classification problem. In many practical domains, the available knowledge is incomplete, the number of labeled examples is small, or the encoded knowledge changes over time. The decisions made by the architecture can thus be incorrect or sub-optimal, e.g., a traffic sign can be misclassified or an ambiguous answer may be provided to an explanatory question. The fourth component of our architecture seeks to address this problem by supporting incremental learning of domain knowledge. Our approach is inspired by the inductive learning methods mentioned in section 2, e.g., Sridharan and Meadows (2018) use relational reinforcement learning and decision tree induction to learn domain axioms. The work described in this paper uses decision tree induction to learn constraints governing domain states. The methodology used in this component, in the context of VQA, is as follows:

1. Identify training examples that are not classified, or are classified incorrectly, using the existing knowledge. Recall that this step is accomplished by the component described in section 3.2, which processes each training example using the existing knowledge encoded in the CR-Prolog program, in an attempt to assign a class label to the example.
2. Train a decision tree using the examples identified in Step-1 above. Recall that this step is also accomplished by the component described in section 3.2.
3. Identify paths in the decision tree (from root to leaf) such that (i) there are a sufficient number of examples at the leaf, e.g., 10% of the training examples; and (ii) all the examples at the leaf have the same class label. Since the nodes correspond to checks on the values of domain features, the paths will correspond to combinations of partial state descriptions and class labels that have good support among the labeled training examples. Each such path is translated into a candidate axiom. For instance, the following are two axioms identified by this approach in the SS domain:
  (7a) $$\begin{array}{l} \neg stable\left(S\right)\;\leftarrow \;num\text{\_}blocks\left(S,3\right),\;base\left(S,wide\right),\\ \;struc\text{\_}type\left(S,lean\right) \end{array}$$
  (7b) $$\begin{array}{l} \neg stable\left(S\right)\;\leftarrow \;num\text{\_}blocks\left(S,3\right),\;base\left(S,narrow\right),\\ \;struc\text{\_}type\left(S,lean\right) \end{array}$$
4. Generalize candidate axioms if possible. For instance, if one candidate axiom is a over-specification of another existing axiom, the over-specified version is removed. In the context of the axioms in Statement 7(a-b), the second literal represents redundant information, i.e., if a structure with three blocks has a significant lean, it is unstable irrespective of whether the base of the structure is narrow or wide. Generalizing over these two axioms results in the following candidate axiom:
  (8) $$\begin{array}{l} \neg stable\left(S\right)\;\leftarrow \;num\text{\_}blocks\left(S,3\right),struc\text{\_}type\left(S,lean\right) \end{array}$$
  which only includes the literals that encode the essential information.
5. Validate candidate axioms one at a time. To do so, the candidate axiom is added to the CR-Prolog program encoding the domain knowledge. A sufficient number of training examples (e.g., 10% of the dataset, as before) relevant to this axiom, i.e., the domain features encoded by the examples should satisfy the body of the axiom, are drawn randomly from the training dataset. If processing these selected examples with the updated CR-Prolog program results in misclassifications, the candidate axiom is removed from further consideration.
6. Apply sanity checks to the validated axioms. The validated axioms and existing axioms are checked to remove over-specifications and retain the most generic version of any axiom. Axioms that pass these sanity checks are added to the CR-Prolog program and used for subsequent reasoning.

Section 4.3 examines the effect of such learned constraints on classification and VQA performance.

### 3.5. Planning With Domain Knowledge

**Figure 7:**
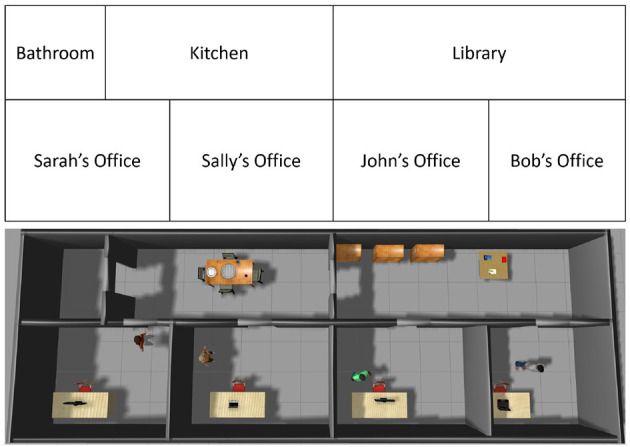
Block diagram and a simulated scenario in the RA domain in which the robot has to deliver messages to people in target locations.

The description of the architecture's components has so far focused on classification and VQA, and reasoning has been limited to inference with knowledge. However, the architecture is also applicable to planning (and diagnostics) problems. Consider the **RA domain** in which a simulated robot has to navigate and deliver messages to particular people in particular places, and to answer explanatory questions, i.e., the domain includes aspects of planning and VQA. Figure 7 depicts this domain and a simulated scenario in it; semantic labels of the offices and rooms are shown in the upper half.

A robot planning and executing actions in the real world has to account for the uncertainty in sensing and actuation. In other work, we addressed this issue by coupling ASP-based coarse-resolution planning with probabilistic fine-resolution planning and execution (Sridharan et al., 2019). In this paper, we temporarily abstract away such probabilistic models of uncertainty to thoroughly explore the interplay between reasoning and learning, including the effect of added noise in sensing and actuation (in simulation).

To support planning, the signature Σ of system description D has basic sorts such as: *place*, *robot*, *person*, *object*, *entity*, *status*, and *step*, which are arranged hierarchically, e.g., *robot* and *person* are subsorts of *agent*, and *agent* and *object* are subsorts of *entity*. Σ also includes ground instances of sorts, e.g., *office*, *workshop*, *kitchen*, and *library* are instances of *place*, and *Sarah*, *Bob*, *John*, and *Sally* are instances of *person*. As before, domain attributes and actions are described in terms of the sorts of their arguments. The fluents include *loc*(*agent, place*), which describes the location of the robot and people in the domain, and *message*_*status*(*message*_*id, person, status*), which denotes whether a particular message has been delivered (or remains undelivered) to a particular person. Static attributes include relations such as *next*_*to*(*place, place*) and *work*_*place*(*person, place*) to encode the arrangement of places and the work location of people (respectively) in the domain. Actions of the domain include:

(9) $$\begin{array}{l} move\left(robot,place\right)\\ deliver\left(robot,message\text{\_}id,person\right) \end{array}$$

which move the robot to a particular place, and cause a robot to deliver a particular message to a particular person (respectively). For ease of explanation, we assume that the locations of people are defined fluents whose values are determined by external sensors, and that the locations of objects are static attributes; as a result, we do not consider actions that change the value of these attributes. The signature Σ also includes (as before) the relation *holds*(*fluent, step*) to imply that a particular fluent is true at a particular time step.

Axioms of the RA domain capture the domain's dynamics. These axioms include causal laws, state constraints and executability conditions encoded as statements in ${\mathrm{AL}}_{d}$ such as:

(10a) $$\begin{array}{l} move\left(rob_{1},L\right)\;\text{causes}\;loc\left(rob_{1},L\right) \end{array}$$

(10b) $$\begin{array}{l} deliver\left(rob_{1},ID,P\right)\;\text{causes}\;message\text{\_}status\left(ID,P,delivered\right) \end{array}$$

(10c) $$\begin{array}{l} loc\left(P,L\right)\;\text{i}\text{f}\;work\text{\_}place\left(P,L\right),not\;\neg loc\left(P,L\right) \end{array}$$

(10d) $$\begin{array}{l} \neg loc\left(T,L_{2}\right)\;\text{i}\text{f}\;loc\left(T,L_{1}\right),L_{1}\neq L_{2} \end{array}$$

(10e) $$\begin{array}{l} \text{impossible}\;deliver\left(rob_{1},ID,P\right)\;\text{if }loc\left(rob_{1},L_{1}\right),loc\left(P,L_{2}\right),\\ \;L_{1}\neq L_{2} \end{array}$$

(10f) $$\begin{array}{l} \text{impossible}\;move\left(rob_{1},L\right)\;\text{i}\text{f}\;loc\left(rob_{1},L\right) \end{array}$$

where Statement 10(a) states that executing a move action causes the robot's location to be the target place; Statement 10(b) states that executing a deliver action causes the message to be delivered to the desired person; Statement 10(c) is a constraint stating that unless told otherwise the robot expects (by default) a person to be in her/his place of work; Statement 10(d) is a constraint stating that any thing can be in one place at time; Statement 10(e) implies that a robot cannot deliver a message to an intended recipient if the robot and the person are not in the same place; and Statement 10(f) states that a robot cannot move to a location if it is already there.

As described in section 3.2, the domain history is a record of observations (of fluents), the execution of actions, and the values of fluents in the initial state. Also, planning (similar to inference) is reduced to computing answer set(s) of the program Π(D,H) after including some helper axioms for computing a minimal sequence of actions; for examples, please see Gelfond and Kahl (2014) and Sridharan et al. (2019). If the robot's knowledge of the domain is incomplete or incorrect, the computed plans may be suboptimal or incorrect. The approach described in section 3.4 can then be used to learn the missing constraints; we will explore the interplay between learning and planning in section 4.4.

## 4. Experimental Setup and Results

In this section, we describe the results of experimentally evaluating the following hypotheses about the capabilities of our architecture:

- **H1:** for the underlying classification problem, our architecture outperforms an architecture based on just deep networks for small training datasets, and provides comparable performance as the size of the dataset increases;
- **H2:** in the context of answering explanatory questions, our architecture provides significantly better performance in comparison with an architecture based on deep networks;
- **H3:** our architecture supports reliable and incremental learning of state constraints, which improves the ability to answer explanatory questions; and
- **H4:** our architecture can be adapted to planning tasks, with the incremental learning capability improving the ability to compute minimal plans.

These hypotheses were evaluated in the context of the domains (SS, TS, and RA) introduced in section 3. Specifically, hypotheses *H*1, *H*2, and *H*3 are evaluated in the SS domain and TS domain in the context of VQA. As stated in section 1, VQA is used in this paper only as an instance of a complex task that requires explainable reasoning and learning. We are primarily interested in exploring the interplay between reasoning with commonsense domain knowledge, incremental learning, and deep learning, in any given domain in which large labeled datasets are not readily available. State of the art VQA algorithms, on the other hand, focus instead on generalizing across different domains, using benchmark datasets of several thousand images. Given the difference in objectives between over work and the existing work on VQA, we thus do not compare with state of the art algorithms, and do not use the benchmark VQA datasets. Furthermore, we evaluated hypothesis *H*4 in the RA domain in which the robot's goal was to deliver messages to appropriate people and answer explanatory questions about this process.

We begin by describing some execution traces in section 4.1 to illustrate the working of our architecture. This is followed by sections 4.2–4.4, which describe the results of experimentally evaluating the classification, VQA, axiom learning, and planning capabilities, i.e., hypotheses H1–H4. We use accuracy (precision) as the primary performance measure. Classification accuracy was measured by comparing the assigned labels with the ground truth values, and question answering accuracy was evaluated heuristically by computing whether the answer mentions all image attributes relevant to the question posed. This relevance was established by a human expert, the lead author of this paper. Unless stated otherwise, we used two-thirds of the available data to train the deep networks and other computational models, using the remaining one-third of the data for testing. For each image, we randomly chose from the set of suitable questions for training the computational models. We repeated this process multiple times and report the average of the results obtained in these trials. For planning, accuracy was measured as the ability to compute minimal and correct plans for the assigned goals. Finally, section 4.5 discusses the reduction in computational effort achieved by our architecture in comparison with the baselines.

### 4.1. Execution Traces

The following execution traces illustrate our architecture's ability to reason with commonsense knowledge and learned models to provide intuitive answers for explanatory questions.

**Execution Example 1**. *[Question Answering, SS domain]* Consider a scenario in the SS Domain in which the input (test) image is the one on the extreme right in Figure 2.

- First **classification-related question** posed: “*is this structure unstable*?”
  The architecture's **answer**: “*no*”.
- The **explanatory question** posed: “*what is making this structure stable*?”
  The architecture's **answer**: “*the structure has five blocks and a narrow base, it is standing straight, and there is no significant lean*”.
- This answer was based on the following features extracted by CNNs from the image: (i) five blocks; (ii) narrow base; (iii) standing straight; and (iv) no significant lean, i.e., all blocks in place.
- The extracted features were converted to literals. ASP-based inference provided an answer about the stability of the arrangement of objects in the scenario. Relevant literals in the corresponding answer set were then inserted into a suitable template to provide the answers described above.
- Since the example was processed successfully using ASP-based inference, it was not processed using the decision tree (for classification) or the RNN (for answering the explanatory question).

**Execution Example 2**. *[Question Answering, TS domain]* Consider a scenario in the TS Domain with the input (test) image is the one on the extreme right in Figure 3.

- The **classification question** posed was: “*what is the sign's message*?”
  The architecture's **answer**: “*uneven surfaces ahead*”.
- When asked to explain this answer (“*Please explain this answer*”), the architecture identified that the CNNs extracted the following features of the sign in the image: (i) it is triangle-shaped; (ii) main color is white and other (i.e., border) color is red; (iii) it has no background image; (iv) it has a bumpy-road symbol and no secondary symbol; and (v) it has no cross.
- These features were converted to literals and used in ASP-based inference based on existing knowledge in the TS domain. ASP-based inference is unable to provide an answer, i.e., unable to classify the sign.
- The extracted features were processed using the trained decision tree, which only used the colors in the sign to assign the class label. The main (or border) color is normally insufficient to accurately classify signs. However, recall that the decision tree is trained to classify signs that cannot be classified by reasoning with existing knowledge.
- The decision tree output, image feature vector, and input question, were processed by the previously trained RNN to provide the answer type and the particular answer described above.

These (and other such) execution traces illustrate the working of our architecture, especially that:

- The architecture takes advantage of (and perform non-monotonic logical inference with) the existing commonsense domain knowledge to reliably and efficiently address the decision-making problem (classification in the examples above) when possible. In such cases, it is also able to answer explanatory questions about the classification decision and the underlying scene.
- When the desired decision cannot be made using non-monotonic logical inference with domain knowledge, the architecture smoothly transitions to training and using a decision-tree to make and explain the classification decision. In such cases, the architecture also learns and uses an RNN to answer explanatory questions about the scene.

### 4.2. Experimental Results: Classification + VQA

**Figure 8:**
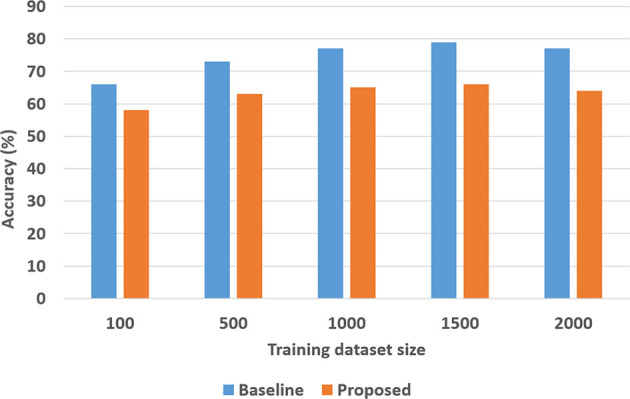
Classification accuracy as a function of the number of training samples in the SS domain.

**Figure 9:**
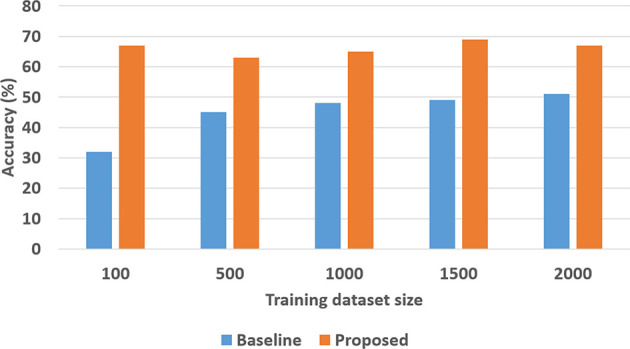
VQA accuracy as a function of the number of training samples in the SS domain.

**Figure 10:**
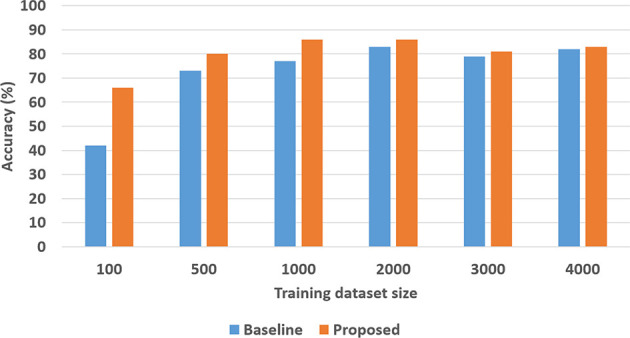
Classification accuracy as a function of number of training samples in TS domain.

**Figure 11:**
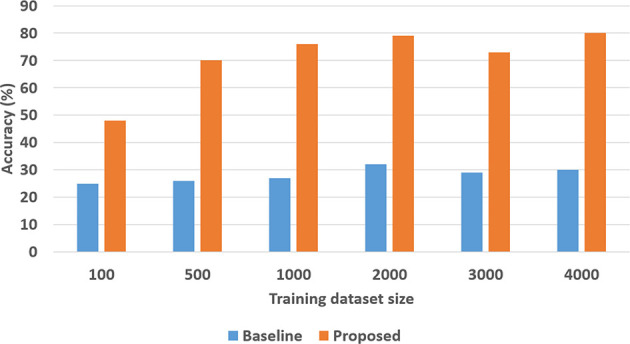
VQA accuracy as a function of number of training samples in TS domain.

To quantitatively evaluate hypotheses *H*1 and *H*2, we ran experimental trials in which we varied the size of the training dataset. In these trials, the baseline performance was provided by a CNN-RNN architecture, with the CNNs processing images to extract and classify features, and the RNN providing answers to explanatory questions. The number of questions considered depends on the complexity of the domain, e.g., we included eight different types of questions in the SS domain and 248 different types of questions in the TS domain. We repeated the trials 50 times (choosing the training set randomly each time) and the corresponding average results are summarized in Figures 8, 9 for the SS domain, and in Figures 10, 11 for the TS domain. We make some observations based on these figures:

1. The classification performance of our architecture depends on the domain. In the relatively simpler SS domain, the baseline deep network architecture is at least as accurate as our architecture, even with a small training set—see Figure 8. This is because small differences in the position and arrangement of blocks (which could almost be considered as noise) influence the decision about stability. For instance, two arrangements of blocks that are almost identical end up receiving different ground truth labels for stability, and it is not possible to draft rules based on abstract image features to distinguish between these cases. The baseline deep network architecture, which generalizes from data, is observed to be more sensitive to these small changes than our architecture. Exploring the reason for this performance is an interesting direction for further research.
2. In the more complex TS domain, our architecture provides better classification accuracy than the baseline architecture based on just deep networks, especially when the size of the training set is small—see Figure 10. The classification accuracy increases with the size of the training set^3^, but our architecture is always at least as accurate as the baseline architecture.
3. Our architecture is much more capable of answering explanatory questions about the classification decisions than the baseline architecture. When the answer provided by our architecture does not match the ground truth, we are able to examine why that decision was made. We were thus able to understand and explain the lower classification accuracy of our architecture in the SS domain. The baseline architecture does not provide this capability.
4. Unlike classification, the VQA performance of our architecture is much better than that of the baseline architecture in both domains. Also performance does not improve just by increasing the size of training set, even in simpler domains, e.g., see Figure 9. This is because VQA performance also depends on the complexity of the explanatory questions. For more complex domains, the improvement in VQA accuracy provided by our architecture is much more pronounced, e.g., see Figure 11.

We explored the statistical significance of the observed performance by running paired t-tests. We observed that the VQA performance of the proposed architecture was significantly better than that of the baseline architecture; this is more pronounced in the TS domain that is more complex than the SS domain. Also, although the baseline architecture provides better classification performance in the SS domain, the difference is not always statistically significant.

To further explore the observed results, we obtained a “confidence value” from the logits layer of each CNN used to extract a feature from the input image. For each CNN, the confidence value is the largest probability assigned to any of the possible values of the corresponding feature, i.e., it is the probability assigned to the most likely value of the feature. These confidence values are considered to be a measure of the network's confidence in the corresponding features being a good representation of the image. We trained a version of our architecture in which if the confidence value for any feature was low, the image features were only used to revise the decision tree (during training), or were processed using the decision tree (during testing). In other words, features that do not strongly capture the essence of the image are not used for non-monotonic logical reasoning; the deep network architectures provide much better generalization to noise. We hypothesized that this approach would improve the accuracy of classification and question answering, but it did not make any significant difference in our experimental trials. We believe this is because the extracted features were mostly good representations of the objects of interest in the images. We thus did not use such networks (that compute the confidence value) in any other experiments.

### 4.3. Experimental Results: Learn Axiom + VQA

**Figure 12:**
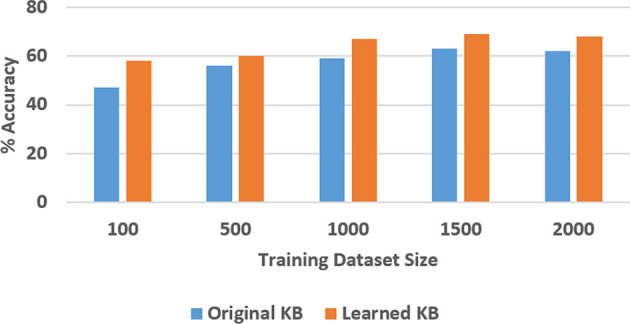
Comparison of classification accuracy in the SS domain with and without axiom learning. In both cases, some axioms were missing from the knowledge base.

**Figure 13:**
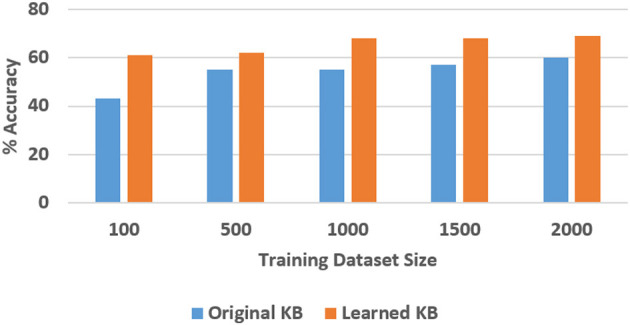
Comparison of VQA accuracy in the SS domain with and without axiom learning. In both cases, some axioms were missing from the knowledge base.

**Figure 14:**
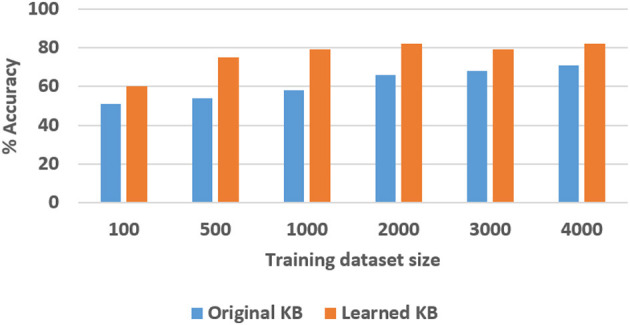
Comparison of classification accuracy in the TS domain with and without axiom learning. In both cases, some axioms were missing from the knowledge base.

**Figure 15:**
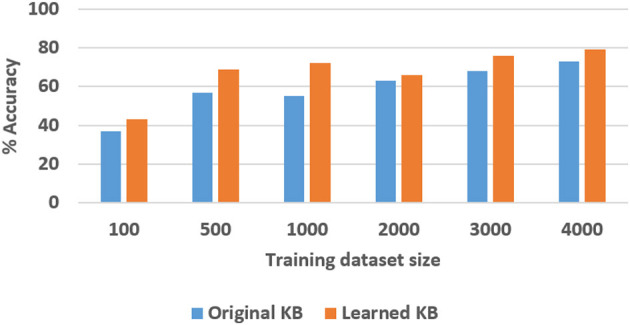
Comparison of VQA accuracy in the TS domain with and without axiom learning. In both cases, some axioms were missing from the knowledge base.

Next, we experimentally evaluated the ability to learn axioms, and the effect of such learning on the classification and VQA performance. For the SS domain, we designed a version of the knowledge base with eight axioms related to stability or instability of the structures. Out of these, four were chosen (randomly) to be removed and we examined the ability to learn these axioms, and the corresponding accuracy of classification and VQA, as a function of the number of labeled training examples (ranging from 100 to 2,000). We repeated these experiments 30 times and the results (averaged over the 30 trials) are summarized in Figures 12, 13. In the TS domain, the methodology for experimental evaluation was the same. However, since the domain was more complex, there were many more axioms in the domain description (for classification and VQA); we also had access to more labeled training examples. In each experimental trial, a quarter of the available axioms were thus selected and commented out, and the accuracy of classification and VQA were evaluated with the number of labeled training examples varying from 100 to 4000. The results averaged over 30 such trials are summarized in Figures 14, 15.

In these figures, “Original KB” (depicted in blue) represents the baseline with some axioms missing from the system description, e.g., four in the SS domain and one quarter of the axioms in the TS domain. The results obtained by using the available labeled examples to learn the axioms that are then used for classification and answering explanatory questions about the scene, are shown as “Learned KB” in orange. We observe that our approach supports incremental learning of the domain axioms, and that using the learned axioms improves the classification accuracy and the accuracy of answering explanatory questions, in comparison with the baseline. This improvement was found to be statistically significant using paired tests at 95% level of significance. These results support hypothesis *H*3.

### 4.4. Experimental Results: Learn Axiom + Plan

Next, we experimentally evaluated the ability to learn axioms and the effect of the learned axioms on planning, in the RA domain. The simulated robot was equipped with domain knowledge for planning, classification, and question answering. It uses this knowledge to navigate through an office building, locate the intended recipient of a message, deliver the message, detect and reason about objects in its surroundings, and answer questions about the rooms it has visited. We considered 24 different types of questions in this domain. As stated in section 3.5, we limit uncertainty in sensing and actuation on robots to noise added in simulation. Average results from 100 trials indicates a VQA accuracy of ≈85% after training the architecture's components with just 500 labeled images. The domain knowledge includes learned axioms—the corresponding experimental results and the planning performance are discussed later in this section. We begin with an execution trace in this domain.

**Execution Example 3**. *[Question Answering, RA Domain]* Consider the scenario in the RA domain (Figure 7) in which the robot's goal was to deliver a message from John to Sally, and return to John to answer questions.

- The robot was initially in John's office. It computed a plan that comprises moving to Sally's office through the library and the kitchen, delivering the message to Sally, and returning to John's office through the same route to answer questions.
- During plan execution, the robot periodically takes images of the scenes in the domain, which are used for planning, classification and question answering.
- After returning to John's office, the robot and the human had an exchange about the plan constructed and executed, and the observations received. The exchange includes instances such as:
  **John's question:** “is Sally's location cluttered?”
  **Robot's answer:** “Yes.”
  When asked, the robot provides an **explanation** for this decision: “Sally is in her office. Objects detected are Sally's chair, desk, and computer, and a cup, a large box, and a sofa. The room is cluttered because the cup, large box, and sofa are not usually in that room.”

The RA domain was also used to evaluate the effects of axiom learning. There were four employees in offices in the simulated scenario, as shown in Figure 7, and the robot was asked to find particular individuals and deliver particular messages to them. Employees are initially expected to be in their assigned workplace (i.e., their office), and spend most of their time in these offices, unless this default knowledge has been negated by other knowledge or observations. This information is encoded as follows:

$$\begin{array}{l} holds\left(loc\left(P,L\right),0\right)\;\leftarrow \;not\;default\text{\_}negated\left(P,L\right),work\text{\_}place\left(P,L\right) \end{array}$$

where *work*_*place*(*P, L*) specifies the default location of each person, and *default*_*negated*(*P, L*) is used to encode that a particular person may not be in their default location. These exceptions to the defaults can be encoded as follows:

(11a) $$\begin{array}{l} default\text{\_}negated\left(P,L\right)\;\leftarrow obs\left(loc\left(P,L1\right),true,I\right),L\neq L1 \end{array}$$

(11b) $$\begin{array}{l} default\text{\_}negated\left(P,L\right)\;\leftarrow obs\left(loc\left(P,L\right),false,I\right) \end{array}$$

Statement 11(a) implies that the default assumption should be ignored if the person in question is observed to be in a location other than their workplace, and Statement 11(b) implies that a default assumption should be ignored if the corresponding person is not observed in their workplace. Including such default knowledge (and exceptions) in the reasoning process allows the robot to compute better plans and execute the plans more efficiently, e.g., when trying to deliver a message to a particular person. However, this knowledge may not be known in advance, the existing knowledge may be inaccurate or change with time (e.g., humans can move between the different places), or the observations may be incorrect. Our axiom learning approach was used in this domain to acquire previously unknown information about the default location of people and exceptions to these defaults. In all the trials, the simulated robot was able to learn the previously unknown axioms.

We then conducted 100 paired trials to explore the effects of the learned axioms on planning, with the corresponding results summarized in Table 1. In each trial, we randomly chose a particular goal and initial conditions, and measured planning performance before and after the previously unknown axioms had been learned and used for reasoning. Since the initial conditions are chosen randomly, the object locations, the initial location of the robot, and the goal, may vary significantly between trials. Under these circumstances, it is not meaningful to average the results obtained in the individual trials for performance measures such as planning time and execution time. Instead, the results obtained without including the learned axioms were computed as a ratio of the results obtained after including the learned axioms; the numbers reported in Table 1 are the average of these computed ratios. Before axiom learning, the robot often explored an incorrect location (for a person) based on other considerations (e.g., distance to the room) and ended up having to replan. After the previously unknown axioms were included in the reasoning process, the robot went straight to the message recipient's most likely location, which also happened to be the actual location of the recipient in many trials. As a result, we observe a (statistically) significant improvement in planning performance after the learned axioms are used for reasoning. Note that in the absence of the learned axioms, the robot computes four times as many plans taking six times as much time in any given trial (on average) as when the learned axioms are included in reasoning. Even the time taken to compute each plan (with potentially multiple such plans computed in each trial) is significantly higher in the absence of the learned axioms. This is because the learned axioms enable the robot to eliminate irrelevant paths in the transition diagram from further consideration. In a similar manner, reasoning with intentional actions enables the robot to significantly reduce the plan execution time by terminating or revising existing plans when appropriate, especially in the context of unexpected successes and failures. These results provide evidence in support of hypothesis *H*4.

**Table 1:** Planning performance in a scenario in the RA domain (see Figure 7) before and after axiom learning.

**Axiom learning**	**Plans** **(per trial)**	**Actions** **(per trial)**	**Execution time** **(per trial)**	**Planning time** **(per trial)**	**Planning time** **(per plan)**
Before	4	2.3	1.6	6.0	1.6
After	1	1	1	1	1

*Results averaged over 100 paired trials indicate that reasoning with previously unknown axioms results in fewer plans with fewer actions in each trial, and significantly reduces the time taken to compute and execute the plans*.

Finally, we conducted some initial proof of concept studies exploring the use of our architecture on physical robots. We considered a robot collaborating with a human to jointly describe structures of blocks on a tabletop (similar to the SS domain described in this paper). We also considered a mobile robot finding and moving objects to desired locations in an indoor domain (similar to the RA domain). These initial experiments provided some promising outcomes. The robot was able to provide answers to explanatory questions, compute and execute plans to achieve goals, and learn previously unknown constraints. In the future, we will conduct a detailed experimental analysis on robots in different domains.

### 4.5. Computational Effort

In addition to the improvement in accuracy of classification and VQA, we also explored the reduction in computational effort provided by our architecture in comparison with the baselines. Measuring this time quantitatively is challenging because it depends on various factors such as the task being performed (e.g., classification, VQA), the knowledge encoded in the knowledge base, the size and order of samples in the training set, and the parameters of the deep networks. However, we were able to gain the following insights.

The computation time includes the training time and the testing (i.e., execution) time, and we first considered the training time. Depending on the task being performed (e.g., classification, VQA, and/or planning), this time includes the time taken to encode and draw inferences from the knowledge base, process queries and construct answers, and train the deep network models. Encoding the incomplete domain knowledge is a one-time exercise for any given domain. The time taken to reason with this knowledge, and the time taken to process queries and construct answers, are negligible in comparison with the time taken to learn the deep network models. Also, the use of CNNs to extract features from images is common to both our architecture and the baselines, and these networks (for the most part) do not need to be retrained multiple times for any given domain. The key difference between our architecture and the baselines is observed in the context of answering explanatory questions about the scenes and the underlying classification problem. Recall that with our architecture, only examples that cannot be processed by ASP-based reasoning are processed by decision-trees and the RNNs for VQA. In our experimental trials, ≈ 10 − 20% of a training set is used (on average) to train the RNNs with our architecture, whereas the entire training set is used for training the RNNs with the baseline architectures. This difference often translates to an order of magnitude difference in the training time, e.g., a few minutes for each training set (in a particular domain) with our architecture compared with hours or days with the baseline architectures. Note that accuracy of our architecture is still much better than that of the baselines, e.g., see Figures 9, 11, i.e., any given accuracy is achieved using a much smaller number of training samples.

The execution time of our architecture is comparable with that of the baselines and is often less. Once the deep network models have been learned, using them for the different tasks does not take much time, e.g., a few seconds to process the input and provide a decision and/or the answer to a query. However, similar to the situation during training, only test samples that cannot be processed by ASP-based reasoning are processed by the decision trees and RNNs with our architecture. Also, since the deep networks in our architecture only need to disambiguate between a small(er) number of training examples, they often have a much simpler structure than the deep networks in the baseline architectures.

Note that in addition to classification and VQA, our architecture also supports explainable reasoning for planning and incremental learning of previously unknown constraints. Providing similar capabilities using just deep network architectures will (at the very least) require a large number of training examples of planning under different conditions; it is often not possible to provide such training examples in dynamic domains. We thus conclude that our architecture significantly reduces the computational effort while supporting a range of capabilities in comparison with the baseline architectures comprising deep networks.

## 5. Discussion and Future Work

Visual question answering (VQA) combines challenges in computer vision, natural language processing, and explainability in reasoning and learning. Explanatory descriptions of decisions help identify errors, and to design better algorithms and frameworks. In addition, it helps improve trust in the use of reasoning and learning systems in critical application domains. State of the art algorithms for VQA are based on deep networks and the corresponding learning algorithms. Given their focus on generalizing across different domains, these approaches are computationally expensive, require large training datasets, and make it difficult to provide explanatory descriptions of decisions. We instead focus on enabling reliable and efficient operation in any given domain in which a large number of labeled training examples may not be available. Inspired by research in cognitive systems, our architecture tightly couples representation, reasoning, and interactive learning, and exploits the complementary strengths of deep learning, non-monotonic logical reasoning with commonsense knowledge, and decision tree induction. Experimental results on datasets of real world and simulated images indicate that our architecture provides the following benefits in comparison with a baseline architecture for VQA based on deep networks:

1. Better accuracy, improved sample efficiency, and reduced computational effort on classification problems when the training dataset is small, and comparable accuracy with larger datasets while still using only a subset of these samples for training;
2. Ability to provide answers to explanatory questions about the scenes and the underlying decision making problems (e.g., classification, planning);
3. Incremental learning of previously unknown domain constraints, whose use in reasoning improves the ability to answer explanatory questions; and
4. Ability to adapt the complementary strengths of non-monotonic logical reasoning with commonsense domain knowledge, inductive learning, and deep learning, to address decision-making (e.g., planning) problems on a robot.

Our architecture opens up multiple directions of future work, which will address the limitations of existing work and significantly extend the architecture's capabilities. We discuss some of these extensions below:

1. The results reported in this paper are based on image datasets (simulated, real-world) chosen or constructed to mimic domains in which a large, labeled dataset is not readily available. One direction of future work is to explore the use of our architecture in other domains that provide datasets of increasing complexity, i.e., with a greater number of features and more complex explanatory questions. This exploration may require us to consider larger datasets, and to examine the trade-off between the size of the training dataset, the computational effort involved in processing such a dataset with many labeled examples, and the effort involved in encoding and reasoning with the relevant domain knowledge.
2. In our architecture, we have so far used variants of existing network structures as the deep network components (i.e., CNN, RNN). In the future, we will explore different deep network structures in our architecture, using the explanatory answers to further understand the internal representation of these network structures. Toward this objective, it would be particularly instructive to construct and explore deep networks and logic-based domain representations that provide similar behavior on a set of tasks, or provide different behavior when operating on the same dataset. As stated in the discussion in section 4.2, such an exploration may help us better understand (and improve) the design and use of deep network models for different applications.
3. This paper used VQA as a motivating problem to address key challenges in using deep networks in dynamic domains with limited labeled training examples. We also described the use of our architecture (with tightly-coupled reasoning and learning components) for planning on a simulated robot. In the future, we will combine this architecture with other architectures we have developed for knowledge representation, reasoning, and interactive learning in robotics (Sridharan and Meadows, 2018; Sridharan et al., 2019). The long-term goal will be to support explainable reasoning and learning on a physical robot collaborating with humans in complex domains.

## Data Availability Statement

The datasets generated or analyzed for this study, and the software implementation of the architecture and algorithms, can be found in the following online repository: https://github.com/hril230/masters_code.

## Author Contributions

HR and MS designed the algorithms and architecture, experimental setup, and analyzed the results. HR implemented the algorithms and architecture with feedback from MS. HR conducted the evaluation. MS wrote the paper with contributions from HR.

### Conflict of Interest

The authors declare that the research was conducted in the absence of any commercial or financial relationships that could be construed as a potential conflict of interest.

## Footnotes

^1^In the RA domain discussed in section 3.5, decision making also includes planning and diagnostics.

^2^An independent group of researchers has developed a general-purposed software to automatically translate any description in ${\mathrm{AL}}_{d}$ to the corresponding CR-Prolog program; we expect this software to be made publicly available soon.

^3^We limit ourselves to training sets that are not too large in order to match the focus of our paper.
